# Imaging-based study demonstrates how the DEK nanoscale distribution differentially correlates with epigenetic marks in a breast cancer model

**DOI:** 10.1038/s41598-023-38685-7

**Published:** 2023-08-07

**Authors:** Agnieszka Pierzynska-Mach, Isotta Cainero, Michele Oneto, Elisa Ferrando-May, Luca Lanzanò, Alberto Diaspro

**Affiliations:** 1 Nanoscopy and NIC @ IIT, Istituto Italiano di Tecnologia, Via Enrico Melen, 83, 16152 Genoa, Italy; 2 Present Address: IRCCS Ospedale Policlinico San Martino, Largo Rosanna Benzi 10, 16132 Genoa, Italy; 3 Department of Biology, University of Konstanz, Konstanz, Germany; 4 German Cancer Research Center, Heidelberg, Germany; 5 Department of Physics and Astronomy, University of Catania, Catania, Italy; 6 DIFILAB, Department of Physics, University of Genoa, Genoa, Italy

**Keywords:** Cellular imaging, Post-translational modifications, Nuclear organization, Breast cancer, Oncogenes, Cancer imaging, Confocal microscopy

## Abstract

Epigenetic dysregulation of chromatin is one of the hallmarks of cancer development and progression, and it is continuously investigated as a potential general bio-marker of this complex disease. One of the nuclear factors involved in gene regulation is the unique DEK protein—a histone chaperon modulating chromatin topology. DEK expression levels increase significantly from normal to cancer cells, hence raising the possibility of using DEK as a tumor marker. Although DEK is known to be implicated in epigenetic and transcriptional regulation, the details of these interactions and their relevance in cancer development remain largely elusive. In this work, we investigated the spatial correlation between the nuclear distribution of DEK and chromatin patterns—alongside breast cancer progression—leveraging image cross-correlation spectroscopy (ICCS) coupled with Proximity Ligation Assay (PLA) analysis. We performed our study on the model based on three well-established human breast cell lines to consider this tumor's heterogeneity (MCF10A, MCF7, and MDA-MB-231 cells). Our results show that overexpression of DEK correlates with the overall higher level of spatial proximity between DEK and histone marks corresponding to gene promoters regions (H3K9ac, H3K4me3), although it does not correlate with spatial proximity between DEK and gene enhancers (H3K27ac). Additionally, we observed that colocalizing fractions of DEK and histone marks are lower for the non-invasive cell subtype than for the highly invasive cell line (MDA-MB-231). Thus, this study suggests that the role of DEK on transcriptionally active chromatin regions varies depending on the subtype of the breast cancer cell line.

## Introduction

The physiological condition of chromatin DNA is maintained based on a complex armada of processes that work in concerted action to ensure DNA regulation. Among all the involved mechanisms, modification of histones represents one of the hallmarks^1^. These modifications—e.g., acetylation, methylation, phosphorylation, and ubiquitination—are often referred to as “the epigenetic code”^2^, since they influence the accessibility of the transcription machinery to DNA without directly altering the DNA sequence. Indeed, the epigenetic code plays a crucial role in orchestrating most DNA-related processes. Non-physiological levels of histone modifications are common in various human diseases—e.g., autoimmune diseases, neurodegenerative diseases, cardiovascular diseases, and cancer^3^—and, as such, are often involved in the prognosis process^4^. For example, in the context of breast cancer, the imbalance of acetylation and methylation of histone tails results in an unusual opening or closing of the chromatin structure^5^, and several other epigenetic marks, e.g. ubiquitination, are highly deregulated^6–12^. In general, the alteration of the normal epigenetic pattern could represent one of the first steps in the process of oncogenic transformation^13^.

Histone modifications regulate gene expression by modulating the spatial accessibility to the genome of various proteins that are involved in the steps of the transcription process—often referred to as the transcription machinery. However, histone modification is not the only mechanism that affects gene regulation: indeed, a variety of proteins plays significant roles to this aim, being involved in the various steps of the transcription process. One of the cellular factors implicated in gene regulation is the DEK protein, also involved in several oncogenic mechanisms^14,15^. DEK protein overexpression is consistently associated with increased cell proliferation, tumor progression, poor prognosis of patients, and, consequently, advanced cancer stage. Moreover, this ubiquitous nuclear factor binds to many highly and commonly expressed genes^16^ and is involved in gene regulation in breast cancer cells^17^. Within this context, it is of great interest to investigate further the interactions of DEK with the open chromatin structure. To do so, we chose multi-color confocal fluorescence microscopy, one of the most powerful and versatile tools to study the spatial co-distribution of nuclear proteins. To increase the information content retrieved by the raw imaging data, we leveraged the pixel-based image cross-correlation spectroscopy (ICCS) analysis, recently applied in a similar context^18–20^. Thanks to the ICCS approach, it is possible to characterize the colocalization of DEK with chromatin markers, thus obtaining an indirect measurement of their functional interactions. To be able to validate these potential interactions, it is often beneficial to complement the imaging colocalization assay with in situ techniques—e.g., Förster Resonance Energy Transfer (FRET) or in vitro co-immunoprecipitation. In our study, we matched the ICCS analysis with the Proximity Ligation Assay (PLA), a promising solution for visualizing the proximity at the nanometer scale between two labeled factors^21,22^. Indeed, this method leverages indirect immunostaining and a subsequent assay involving enzymes to reveal the interactions of endogenous proteins lying less than 40 nm apart^23^.

Here, we applied the ICCS approach and PLA analysis to investigate the spatial correlation between the nuclear distribution of DEK protein and the chromatin pattern in the context of breast cancer progression. We analyzed the landscape of DEK in combination with three prominent histone marks, H3K4me3 and H3K9ac typically associated with transcriptionally active gene promotors, and H3K27ac found at gene enhancers, to quantify their relative spatial distributions. We performed our study leveraging a well-established model of breast cancer progression, i.e., three cell lines which show heterogeneity of this tumor: (i) non-tumorigenic human breast epithelial cell line MCF10A, widely used to study normal breast cell function and transformation^24,25^; (ii) the MCF7 cell line, non-invasive and poorly aggressive, exhibiting low metastatic potential^26^, and often used to represent the mature luminal subtype expressing both the estrogen and progesterone receptors (ER+, PR+); and (iii) the invasive metastatic basal-like cell line, MDA-MB-231, which represents the breast cancer type most difficult to treat: the triple-negative adenocarcinoma (ER-, PR-, HER2-) prone for the epithelial- to mesenchymal transition (EMT)^27,28^.

## Results and discussion

**Figure 1:**
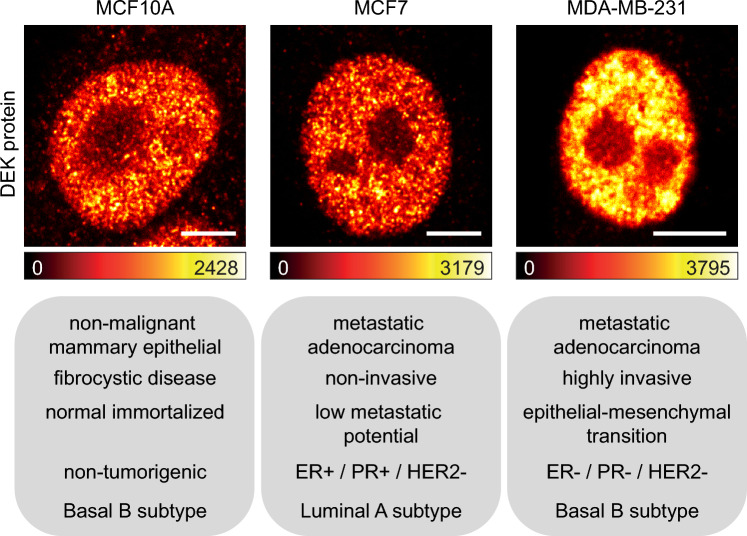
DEK protein confocal imaging in model breast cancer cell lines. DEK protein nuclear distribution in MCF10A, MCF7 and MDA-MB-231 cells representing non-malignant, metastatic non-invasive and metastatic highly invasive state of cancer respectively. Lookup table (LUT) below each image indicates the minimum and maximum grey value of 12 bits image. Scale bars = 5 μm.

First, we evaluated the nuclear distribution of DEK protein among the three chosen breast cancer cell lines using fluorescence confocal microscopy (Fig. 1). Similar to other microscopy studies^15,29–31^, we observed the expected nuclear pattern of DEK in each cell lines: the fluorescence signal appeared uniform within the nucleus, reduced in intensity at its periphery, and absent in nucleoli. In MDA-MB-231 cells, the higher fluorescence intensity—compared with MCF10A and MCF7 cells—revealed the DEK overexpression, typical of more advanced stages of cancer progression^32^.

A growing number of studies implies a connection between altered post-translational modifications of proteins and breast cancer progression^9,11,33,34^. Therefore, for further analysis, we have chosen three post-translational histone modifications which occurrence level is associated with breast cancer: (i) H3K9ac, a histone mark of active gene promoters; (ii) H3K27ac, a well-established mark for active enhancers^35^, or super-enhancers^36,37^; and (iii) H3K4me3, a histone mark of active gene promoters. Literature demonstrates the association of the Western Blot low/moderate detection levels for the H3K9ac modification with poor prognosis^10^, even if this histone mark often correlates with a particular gene implicated in cancer progression^38^. The occurrence of H3K27ac instead is thought to increase in tumor breast tissues^9^. Moreover, for H3K4me3, a high intensity of nuclear H3K4me3 immunohistochemistry staining was found to be correlated with a lower 10-year-survival and with lower breast cancer-specific survival^8^.

**Figure 2:**
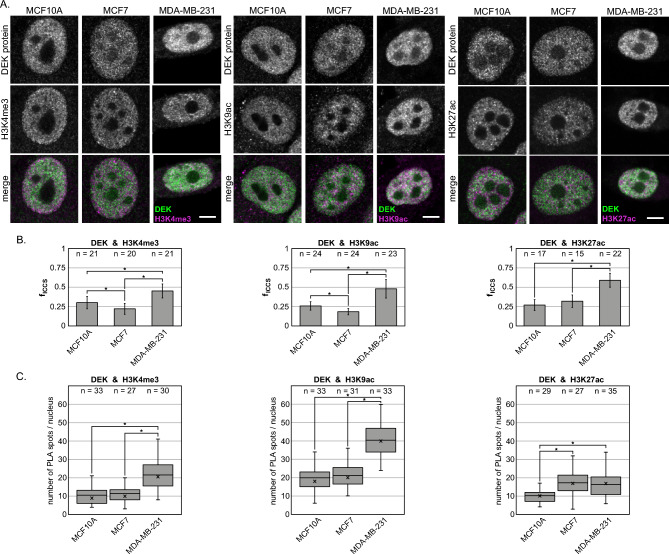
Correlation between DEK protein distribution and active chromatin regions in human breast cancer model. (**A**) Confocal images of co-immunostained breast cancer model cell lines with DEK protein and H3K4me3, H3K9ac or H3K27ac histone modifications. Scale bars = 5 μm. (**B**) Column bar graphs representing means of colocalized fractions extracted from ICCS analysis. (**C**) Box plot graphs of the PLA spots quantitative analysis. For (**B**) and (**C**) statistical significance between all groups (p < 0.0167, marked with an asterisk symbol) was determined by one-way ANOVA with Bonferroni post-hoc test.

In our model, we investigated the spatial colocalization of each of the chosen post-translational modifications and the DEK protein to shine new light on their potential functional relations and, overall, on the role of DEK in breast cancer development. To do so, we took advantage of the imaging-based approaches and leveraged both the image cross-correlation spectroscopy (ICCS) analysis and the proximity ligation assay (PLA) methods. The ICCS has previously been applied to detect dynamic interactions not only relying on confocal imaging but also in the context of super-resolution microscopy such as SIM^39^ or SMLM^20^. PLA instead gives the wide possibility for qualitative testing of the existence of nanoscale proximity and potential protein–protein interactions in an immuno-based experimental design^22,40,41^. Our ICCS analysis of two-colour confocal images (Fig. 2A) revealed that the relative distribution of DEK correlated with euchromatin sites (Fig. 2B). In particular, we observed that the colocalized fraction of DEK with each of the studied H3 modifications was significantly and consistently higher for the invasive metastatic MDA-MB-231 cell line when compared to the other cell lines. The abundance of DEK at these active chromatin marks may suggest the need for DEK recruitment to the gene transcription sites in case of metastatic and invasive breast cancer phenotype. The one-way ANOVA test of the ICCS values showed significant differences between the three cell lines concerning the colocalization of DEK with H3K9ac and H3K4me3 but not H3K27ac. Interestingly, for H3K4me3 and H3K9ac, the colocalized fraction with DEK is lower in MCF7 cells than in MCF10A cells. This result may be explained considering that specific acetylation or methylation events on histone H3 in diverse subtypes of breast cancer are regulated differently depending on the target gene, as shown recently^38^. For H3K27ac and DEK, the average colocalization fraction in MDA-MB-231 cells (0.59 ± 0.09) was significantly higher compared to the average colocalization fraction in MCF10A (0.26 ± 0.06, p < 0.0167) and MCF7 cells (0.30 ± 0.08 p < 0.0167). For the colocalization of H3K27ac and DEK, there was no statistically significant difference between MCF10A and MCF7 cells (p = 0.18). Additionally, global gene expression profiling of breast tumor cell lines shows significant differences between cancer subtypes in the level of expressed genes^42^. In particular, enhancer-associated chromatin states are the most variable across different breast cancer cell lines^7^.

The biological effects of proteins on the cellular state are related not only to their different expression levels but also to the altered interaction with other cellular factors. Therefore, we monitored the potential protein–protein interactions between DEK and euchromatin histone marks listed above. In order to do so, we performed PLA, which recognizes the nanometer-range proximity—and hence the potential interactions—between immunostained molecules. In particular, we analyzed the PLA-detected spots from Z-stack confocal images. The location of PLA foci within the cell nucleus indicates that they form within lower DNA density chromatin regions (Suppl. Fig. 1). After performing a one-way ANOVA test, we observed that there was a statistically significant difference between the average number of PLA spots in the three studied cell lines (Fig. 2C). For DEK and H3K4me3, as well as DEK and H3K9ac, we observe about two times higher numbers of PLA spots in MDA-MB-231 cells than in MCF10A and MCF7 cells. Instead, there was no significant difference between the average of the number of PLA spots between MCF10A cells and MCF7 cells (for H3K9ac and DEK, p = 0.485, for H3K4me3 and DEK, p = 0.498). These results—validated by a set of positive and negative controls (Suppl. Fig. 2)—confirm and enrich our ICCS analysis: in MDA-MB-231 cells, DEK protein localizes in the proximity of H3K4me3 and H3K9ac, and seems to interact with euchromatin marks more than in non-malignant MCF10A or non-invasive MCF7 cells. Interestingly, the PLA analysis shows that—for MDA-MB-231 cells—the molecular proximity between DEK and H3K9ac is significantly higher (40.5 ± 8.9) (Fig. 2C) compared to the MCF10A (19.9 ± 7.9, p < 0.0167) and MCF7 cells (21.1 ± 5.9, p < 0.0167). This suggests the particular importance of DEK on the gene sequences marked by the acetylation of histone H3 on lysine 9. It would be of great interest to investigate further which particular gene promoter displays this epigenetic profile and how it could be regulated by the presence of DEK protein in its proximity.

In MDA-MB-231 cells, DEK is known to be overexpressed^32,43^, and exhibits a higher fluorescence intensity on confocal images than in MCF10A or MCF7 cells. ICCS analysis showed that in MDA-MB-231 cells DEK colocalizes with H3K27ac to a significantly higher degree than in other two cell lines. However, the number of PLA spots does not reflect this trend. The statistical analysis showed no significant difference between MCF7 and MDA-MB-231 cells (p = 0.645) regarding the average number of PLA spots specific for DEK and H3K27ac. Interestingly, this number was lower in MCF10A cells (10.1 ± 4.3) as compared to MCF7 (17.3 ± 5.5, p < 0.0167) and MDA-MB-231 cells (16.5 ± 6.6, p < 0.0167). Although both proteins in MDA-MB-231 cells spatially colocalize (f_ICCS_ = 0.59 ± 0.09), the last results may indicate that they do not stay in proximity lower than 40 nm, suggesting that they do not interact. Thanks to the combination of our microscopy-imaging based approaches, we determined the interactions and spatial proximity between the proteins in intact cells, which could be complemented in the future with the chromatin immunoprecipitation experiments. Based on our study, we can, however hypothesize that DEK is less present around the gene enhancers (or super-enhancers) in this breast cancer subtype.

## Conclusions

In this study, we analyzed the spatial correlation between DEK and euchromatin marks in the heterogeneous breast cancer model composed of MCF10A, MCF7, and MDA-MB-231 cell lines. Specific cross-correlation function signatures were highlighted and complemented with the PLA analysis. Our microscopy imaging-based data show that DEK protein overexpression correlates with a higher level of spatial proximity between DEK and H3K4me3 and between DEK and H3K9ac but does not correlate with this feature between DEK and H3K27ac. We hypothesize that this could be related to the potential increased activity of DEK on gene promoters. Together, these results emphasize the molecular connection between DEK protein and the regulation of active sites of chromatin in the breast cancer model. The novelty of this study lies in the evidence that DEK preferentially stays in proximity to gene promoter regions rather than gene enhancers. In conclusion, the results from this study relate to the many roles of the DEK protein and, in general, pave the road for further investigations of the epigenetic mechanisms of cancerogenesis.

## Materials and methods

### Cell culture

Mammary epithelial, non-transformed MCF10A cells (ATCC CRL-10317) were grown in DMEM:F-12 (Dulbecco's Modified Eagle Medium:Nutrient Mixture F-12) (1:1) medium (Gibco, 11330057) supplemented with 5% Horse Serum (HS), 2 mM l-glutamine and 1% penicillin/streptomycin (Sigma-Aldrich, G6784), 10 µg/ml insulin (Sigma-Aldrich, I9278) and 0.5 µg/ml hydrocortisone (Sigma-Aldrich, H0888). Human Epidermal Growth Factor (hEGF, Sigma-Aldrich, E9644) was added freshly before the use of full medium (20 nm/ml). MCF7 cells and MDA-MB-231 cells (ATTC HTB-26) were grown in DMEM medium (Gibco, 11330057) supplemented with 10% Fetal Bovine Serum (FBS, Euroclone, ECS0180L), 1% penicillin/streptomycin (Sigma-Aldrich, G6784) and 2 mM l-glutamine. Cells were grown on 10 cm^2^ petri dishes for a month (about 10–12 cell passages) at 37 °C in 5% CO_2_. For any experiments on fixed cells, cells were plated on glass coverslips coated with 0.5% (w/v) pork gelatin (Sigma-Aldrich, G2500) previously dissolved in phosphate buffer saline (PBS) and autoclaved.

### Immunolabelling

For the detection of the protein of interest, the cell cultures were washed with pre-warmed PBS 3 times, and fixed with formaldehyde (3.7%, methanol free) for 15 min. After the subsequent blocking with the blocking buffer composed of 0,1% Triton X-100 and 3% w/v of bovine serum albumin (BSA) for 1 h at room temperature, samples were incubated with the following primary (overnight at 4 °C) and secondary antibodies (1 h at room temperature in the dark): α-DEK (mouse, Santa Cruz, sc-136222, 1:50), α-H3K9ac (rabbit, Invitrogen, 710293, 1:250), α-H3K4me3 (rabbit, Abcam, Ab8580, 1:250), α-H3K27ac (rabbit, Invitrogen, 720096, 1:500), α-PCNA (rabbit, Sigma-Aldrich, HPA030522, 1:50), goat α-mouse Alexa Fluor 488 (Invitrogen, A11001, 1:250), goat α-rabbit Atto 532 (Rockland, 611-153-122, 1:200). After the incubation with secondary antibodies, cells were rinsed 3 × with PBS, and incubated for 20 min at RT with TO-PRO™-3 Iodide (Invitrogen, 1:2000) when necessary, and again rinsed 3 × with PBS. The samples were mounted in ProLong™ Diamond Antifade Mountant (Invitrogen, P36961). The positive control for the ICCS analysis was performed on a couple of two DNA replication marks: EdU Click-iT (with Alexa Fluor 594) stained replication foci and PCNA replication protein.

### Proximity ligation assay

After the fixation and permeabilization of cells, samples were processed for the Proximity Ligation Assay (PLA) according to the manufacturer’s instructions (Sigma-Aldrich) using the DuoLink^®^ in situ Orange detection reagent (DUO92102). First, the sample was incubated for 60 min at 37 °C in a humidified chamber in PLA blocking solution. Then the cells were incubated for 1 h at RT with primary antibodies in Antibody Solution: anti-DEK mouse antibody (Santa Cruz, sc-136222, 1:50) and α-H3K9ac (rabbit, Invitrogen, 710293, 1:250), α-H3K4me3 (rabbit, Abcam, Ab8580, 1:250), α-H3K27ac (rabbit, Invitrogen, 720096, 1:500), α-H3K9me2 (mouse, Abcam, Ab1220, 1:200), α-H3K9me3 (rabbit, Abcam, Ab8898, 1:500). After subsequent washing with the Washing Buffer A, we performed the Ligation step for 30 min at 37 °C and Amplification step for 100 min at 37 °C in the dark. After the wash with Washing Buffer B and PBS, the cells were incubated with the secondary antibody anti-mouse Alexa Fluor 488 (ThermoFisher, A11001, 1:250), incubated for 20 min at RT with TO-PRO™-3 Iodide (Invitrogen, 1:2000) when necessary, and washed with PBS. All the samples were mounted in ProLong™ Diamond Antifade Mountant.

### Microscopy imaging

Measurements were performed on a Leica TCS SP5 confocal laser-scanning microscope, using an HCX PL APO 100 ×/1.40–0.70 oil immersion objective lens (Leica Microsystems, Mannheim, Germany). Excitation was provided with a white light laser (SuperK, NKT) at the desired wavelength for each dye: 488 nm (for Alexa Fluor 488), 532 nm (for Atto 532), 554 nm (for Quasar 570), 642 nm (for ToPro3), with relevant emission detection bands 495–525 nm (for Alexa Fluor 488), 540–620 nm (for Atto 532), 560–625 nm (for Quasar 570) and 650–700 nm (for ToPro3). Signals in different channels were detected with photomultipliers (PMT) or Hybrid Detectors (HyD). In most cases, 512 × 512 pixel images were acquired with a pixel size 40 nm.

### Image and statistical analysis

Confocal microscopy images were processed using ImageJ (http://imagej.nih.gov/ij/). Contrast of the images was adjusted to show weaker fluorescence signals. The number of PLA spots was obtained using a plugin 3D Object Counter from Z-stack confocal images.

For the 2D image cross-correlation spectroscopy (ICCS) analysis, we applied the open-source MatLab code developed by the authors of the original article^18^ to series of confocal microscopy images. Briefly, ICCS analysis refers to the spatial variant of the fluorescence correlation spectroscopy (FCS)^44,45^ and fluorescence cross-correlation spectroscopy (FCCS)^46^. ICCS applies the same formalism to the analysis of the spatial intensity fluctuations between two signals in the cell. The ICCS algorithm retrieves the colocalized fraction (f_ICCS_) from the fitting of the image auto- and cross-correlation functions^47,48^. The colocalized fraction retrieved by the ICCS is foci density independent making it advantageous over object-based approaches, particularly when targeting high-density foci^18^. The two-dimensional (2D) correlation functions were calculated as:

1 $$G_{i,j} \left( {\delta_{x} ,\;\delta_{y} } \right) = \frac{{\left\langle {I_{i} \left( {x,\;y} \right)I_{j} \left( {x + \delta_{x} ,\;y + \delta_{y} } \right)} \right\rangle }}{{\left\langle {I\left( {x,\;y} \right)} \right\rangle ^{2} }} - 1$$

where I_1_(x,y) and I_2_(x,y) are the images in the first and the second channel, respectively, and the angle brackets indicate averaging over all the selected pixels of the image. The two autocorrelation functions were obtained by setting i = j = 1 and i = j = 2, respectively, whereas the cross-correlation function was obtained by setting i = 1 and j = 2. The 2D correlation functions were then converted into radial one-dimensional correlation functions G_ij_(δ_r_) by performing an angular mean, as described previously^49^. The resulting radial correlation functions were then fitted to a Gaussian model:

2 $$G_{ij} (\delta_{r} ) = G_{ij} (0)e^{{ - \frac{{\delta_{r}^{2} }}{{w_{ij}^{2} }}}} + G_{ij} (\infty )$$

where *G*_*ij*_(0) is the amplitude parameter, *w*_*ij*_ is a width parameter, *G*_*ij*_(∞) is an offset. Finally, the colocalization fraction *f* was calculated as f = [G_12_(0)/G_22_(0) + G_12_(0)/G_11_(0)]/2.

In order to test if there are statistically significant differences of the means between cell lines (of the number of PLA spots and ICCS average values), the one-way ANOVA test was performed. The differences of variance values were calculated performing *F*-test, followed by two-sample Student's *t*-tests for each investigated couple data set, with the assumption of the two-tailed distribution and homoscedastic or heteroscedastic variance accordingly. In order to avoid the type I error of the *t*-test, we performed the Bonferroni correction for the p values^50^.

### Supplementary Information

Supplementary Figures.

## Data Availability

The datasets used and/or analysed during the current study available from the corresponding author on reasonable request.

## Abbreviations

ICCS: Image cross-correlation spectroscopy
FRET: Förster resonance energy transfer
PLA: Proximity ligation assay
ANOVA: Analysis of variance
FCS: Fluorescence correlation spectroscopy
FCCS: Fluorescence cross-correlation spectroscopy
