# Supplementary material for: Imaging-based study demonstrates how the DEK nanoscale distribution differentially correlates with epigenetic marks in a breast cancer model
Source: Sci Rep. 2023 Aug 7;13:12749. doi: 10.1038/s41598-023-38685-7 (PMC10406876; doi:10.1038/s41598-023-38685-7)
Supplement: Supplementary file 1 — Supplementary Figures. [file 41598_2023_38685_MOESM1_ESM.pdf]

Supplementary material:

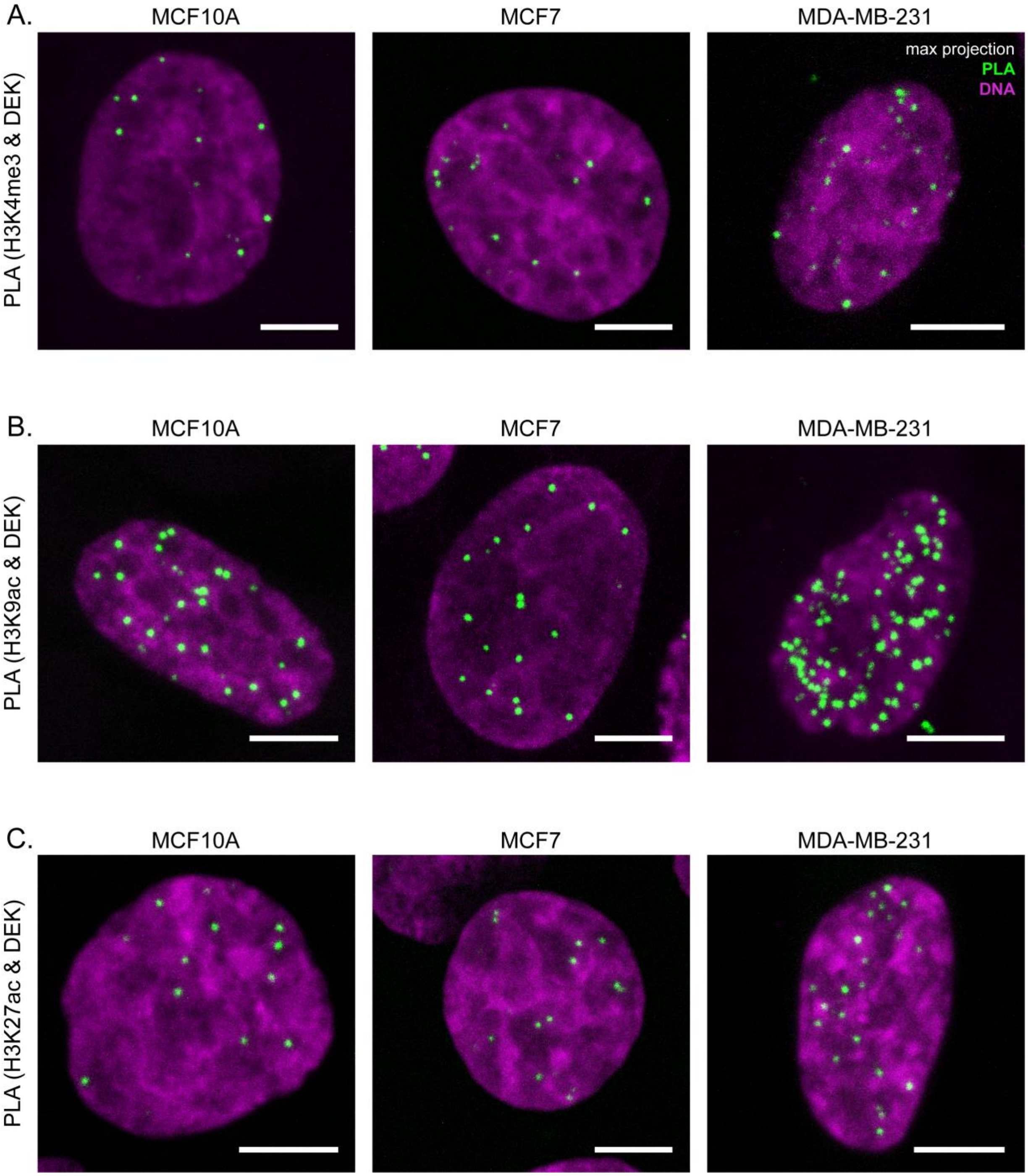

Suppl. Fig. 1. Proximity Ligation Assay between DEK protein and active histone marks. Representative maximum projections from confocal microscopy z-stacks of MCF10A, MCF7 and MDA-MB-231 cells with the DNA stained by ToPro3 (magenta) and PLA (green) between: (A) H3K4me3 and DEK, (B) H3K9ac and DEK, and (C) H3K27ac and DEK. Scale bars = 5 μm.

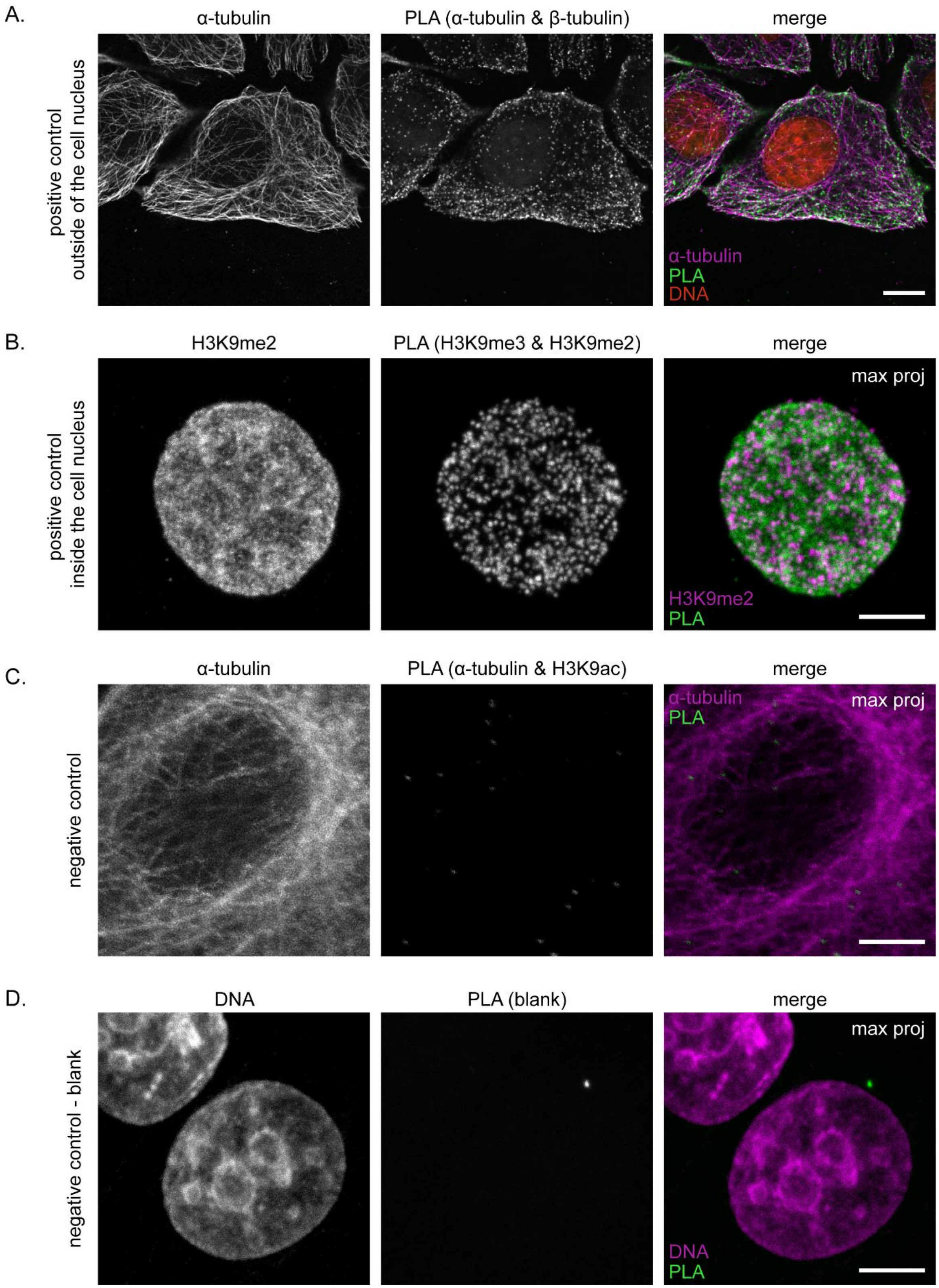

Suppl. Fig. 2. Proximity Ligation Assay control results in MCF10A and MCF7 cells. (A) Positive control of the PLA outside of the cell nucleus; PLA between α-tubulin and β-tubulin, α-tubulin

immunostained with Alexa 488, DNA staining with ToPro3 iodide. Scale bar = 10 µm. (B) Positive control within the cell nucleus; PLA between H3K9me3 and H3K9me2, H3K9me2 immunostained with Alexa 488. Average number of PLA spots = 255.3 ± 62; number of cell nuclei = 23. Scale bar = 5 µm. (C) Negative control; PLA between H3K9ac and α-tubulin, α-tubulin immunostained with Alexa 488. Average number of PLA spots = 6.2 ± 2.4; number of cell nuclei = 19. Scale bar = 5 µm. (D) Negative blank control; No primary antibodies; DNA staining with ToPro3 iodide. Average number of PLA spots = 0.2 ± 0.4; number of cell nuclei = 10. Scale bar = 5 µm.
